# Exploring the Link Between RSV Infection and Antibiotic Prescriptions in Older Adults: A Systematic Review

**DOI:** 10.3390/antibiotics15050514

**Published:** 2026-05-19

**Authors:** Farzaneh Eslami, Priscilla Anyimiah, Sjoukje van der Werf, Maarten J. Postma, Cornelis Boersma

**Affiliations:** 1 Unit of Global Health, Department of Health Sciences, University Medical Center Groningen (UMCG), University of Groningen, 9713 GZ Groningen, The Netherlands; p.anyimiah@umcg.nl (P.A.); m.j.postma@rug.nl (M.J.P.); cornelisboersma@health-ecore.com (C.B.); 2 Health-Ecore, 3704 HE Zeist, The Netherlands; 3 Health-Ecore, 9728 JT Groningen, The Netherlands; 4 Medical Library, University Medical Center Groningen (UMCG), University of Groningen, 9713 AV Groningen, The Netherlands; s.van.der.werf@umcg.nl; 5 Department of Economics, Econometrics and Finance, Faculty of Economics & Business, University of Groningen, 9747 AJ Groningen, The Netherlands; 6 Division of Pharmacology & Therapy, Faculty of Medicine, Universitas Airlangga, Surabaya 60115, Indonesia; 7 Center of Excellence for Pharmaceutical Care Innovation, Universitas Padjadjaran, Bandung 45363, Indonesia; 8 Department of Management Sciences, Open University, 6419 AT Heerlen, The Netherlands

**Keywords:** respiratory syncytial virus, antibiotic prescribing, older adults, antimicrobial stewardship, bacterial co-infection, antimicrobial resistance, respiratory infections, systematic review

## Abstract

Background/Objective: Respiratory syncytial virus (RSV) is an often under-recognized cause of respiratory illness in older adults. Clinical overlap with bacterial infections and delayed virologic confirmation may lead to the unnecessary prescription of antibiotics and antimicrobial resistance (AMR). This systematic review was conducted to assess antibiotic prescription in older adults with RSV and the factors influencing these decisions. Methods: This systematic review was preregistered in PROSPERO (CRD42024586905) and reported according to PRISMA guidelines. PubMed/MEDLINE, Embase, Web of Science, Cochrane CENTRAL, and Scopus were searched for studies published between January 2000 and August 2025. Eligible studies were those including adults aged ≥60 or ≥65 years with RSV infection and reporting antibiotic use. Data on antibiotic prescription, confirmed bacterial infection, hospitalization, length of stay (LOS), and prescribing indications were extracted. Results: Eight observational studies across inpatient, outpatient, emergency, and primary-care settings were included. Antibiotic prescribing ranged from 40.0% to 97.7%, whereas confirmed bacterial infection did not exceed 20% in any study. Antibiotic prescribing was associated with diagnostic uncertainty, radiologic findings, inflammatory markers, respiratory distress, delayed RSV testing, and multimorbidity rather than microbiological confirmation. Hospitalization rates varied across settings, and the LOS ranged from 3.5 to 11 days. None of the studies reported antibiotic discontinuation following RSV confirmation. Conclusions: Older adults with RSV frequently receive antibiotics despite low rates of confirmed bacterial infection, indicating substantial empirical prescribing. Improved rapid diagnostics, reassessment of therapy, and strengthened antimicrobial stewardship may help reduce unnecessary antibiotic use. RSV vaccination may be a promising strategy for reducing severe disease and hospitalization, with a potential indirect effect on antibiotic use, although these effects remain hypothetical.

## 1. Introduction

Respiratory syncytial virus (RSV), a single-stranded RNA virus from the Paramyxoviridae family, is a leading cause of lower respiratory tract infections, and is particularly severe in older individuals from late autumn to early spring [1,2,3,4,5]. In the United States, it leads to approximately 14,000 deaths and 177,000 hospitalizations of adults aged 65 and older each year, imposing a significant health burden [6,7]. In long-term care settings, RSV infection rates can range from 12% to 89%, making institutionalized older adults especially vulnerable [1,6].

The risk of severe RSV-associated acute respiratory illness is particularly elevated among older adults with chronic cardiopulmonary diseases or weakened immune systems [1,8]. Studies also indicate that severe RSV infection can lead to higher morbidity and mortality rates in older adults compared to severe influenza [1,9,10,11]. Despite this burden, RSV is frequently unrecognized in routine clinical practice for this age group. This leads to suboptimal management strategies, including potentially unnecessary antibiotic prescriptions due to misdiagnoses of suspected bacterial infections [5,6,12,13,14,15,16,17]. It has been suggested that RSV contributes substantially to primary care antibiotic prescribing, with many prescriptions likely unnecessary given the viral nature of RSV, the self-limiting nature of most RSV infections, and the absence of bacterial co-infections [18,19]. Older adults have the highest rates of antibiotic prescription by general practitioners, making them a priority group for improving prescribing practices [20,21,22].

The lack of rapid, reliable diagnostic tests for RSV further complicates clinical decision-making, particularly in older adults who often present with nonspecific symptoms [12,23]. During RSV season, antibiotic prescribing rates tend to rise, likely reflecting diagnostic uncertainty rather than confirmed bacterial infection [24,25]. Similar to patterns seen in other respiratory viral infections, such as influenza and COVID-19, empirical antibiotic use is common in RSV cases and often initiated before confirming viral etiology or excluding bacterial infection [26,27]. Because RSV symptoms can closely mimic bacterial pneumonia or other complications, antibiotics are often prescribed even in the absence of microbiological evidence [13,14,15,16,17,26]. These prescriptions are often driven by diagnostic uncertainty and the perceived risk of complications in older adults, resulting in unnecessary antibiotic use and worse outcomes [28]. Clinicians may also continue antibiotics after confirming a viral infection, despite no bacterial evidence [27]. Clearer differentiation between viral and bacterial respiratory infections is essential to reduce potentially unnecessary antibiotic use, which has a role in antimicrobial resistance. Improving access to rapid diagnostics and raising clinical awareness, especially in general practice, are key steps to achieving this goal [29]. The use of antibiotics in viral infections often provides no clinical benefit and is widely recognized as an important contributor to the global threat of antimicrobial resistance (AMR) [30,31]. Reducing unnecessary antibiotic use in older adults with RSV is therefore beneficial not only for patient safety but also to help slow the spread of resistant infections. Older adults are especially vulnerable to the effects of both RSV and AMR due to reduced immune function, underlying health conditions, and frequent hospital exposure [28,32]. Unnecessary antibiotic use in this population can increase the risk of adverse drug reactions, hospital complications, and antibiotic-resistant infections [28,33].

As the global demographic shifts towards an older population, the burden of RSV-related illness is projected to increase, emphasizing the urgent need for targeted preventive and therapeutic strategies [34]. Although treatment options for older adults are limited, new vaccines have recently been approved for individuals aged 60 and older, such as the adjuvanted RSVPreF3 (Arexvy, GSK) and RSVpreF (Abrysvo, Pfizer) vaccines and the mRNA-based RSV vaccine (mRESVIA, Moderna). These vaccines are not routinely used across age groups, highlighting the ongoing need for the further development and widespread deployment of preventive and correct diagnostic tools to reduce RSV complications and unnecessary antibiotic use, especially in vulnerable populations [33,35,36]. This systematic review was conducted to assess the prevalence of antibiotic prescriptions in older adult patients with RSV, examine the clinical and diagnostic factors influencing these decisions, and evaluate how often antibiotics are prescribed in the absence of microbiologically confirmed bacterial co-infection. By synthesizing current evidence, this study can inform clinical practice and support the development of more effective guidelines for managing RSV in older adults while also addressing the downstream impact on antimicrobial resistance.

## 2. Results

### 2.1. Study Selection Process

**Figure 1:**
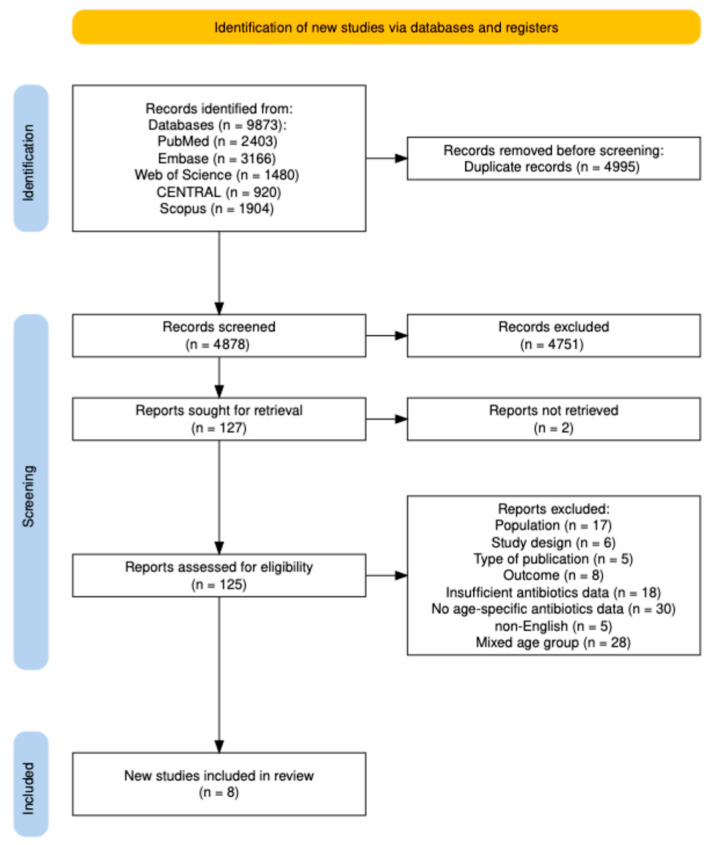
The Prisma flow diagram of the study selection process.

The final literature search was conducted on 25 August 2025 across PubMed, Embase, Web of Science, Cochrane CENTRAL, and Scopus, yielding 9873 records. After the removal of 4995 duplicate records, 4878 unique records remained for title and abstract screening. Of these, 4751 records were excluded. A total of 127 reports were sought for retrieval, of which two could not be obtained. Consequently, 125 full-text articles were assessed for eligibility, and eight studies met the inclusion criteria and were included in the final systematic review. The study selection process is illustrated in the PRISMA 2020 flow diagram below (Figure 1).

### 2.2. Characteristics of the Selected Studies

The characteristics of the included studies are summarized in Table 1. The eight studies were published between 2018 and 2025 and focused exclusively on adults aged ≥60 (*n* = 5) or ≥65 years (*n* = 3). The study designs included retrospective and prospective observational cohorts treated in a range of clinical settings, including inpatient wards, emergency departments, outpatient clinics, and primary care. The studies represented diverse geographic regions from across Europe and North America and varied substantially in sample size. Most studies confirmed RSV infection using molecular diagnostic techniques such as RT-PCR or multiplex PCR, which provide high sensitivity and specificity for viral detection; one large administrative database study relied on diagnostic coding, reflecting clinically diagnosed RSV rather than laboratory-confirmed infection [37]. The mean participant age across studies was between 72 and 85 years.

### 2.3. Clinical Settings of Included Studies

As summarized in Table 1, the included studies were conducted across diverse clinical settings such as inpatient wards, emergency departments, outpatient clinics, and primary care settings. Two studies enrolled mixed inpatient–outpatient cohorts [40,42], while others focused exclusively on hospital inpatients [9,39] or outpatient/community cohorts (Table 1).

### 2.4. Hospitalization and Length-of-Stay (LOS)

Hospitalization and LOS outcomes are summarized in Table 2. Hospital admission rates varied widely across studies, ranging from 3.0% in primary care settings to 100% in studies exclusively targeting hospital inpatients. Inpatient-focused studies, by definition, reported high admission proportions, whereas community and primary care cohorts demonstrated substantially lower hospitalization rates.

Among hospitalized patients, reported LOS varied across cohorts and settings. The median LOS ranged from 3.5 days in outpatient/community settings to 11 days in inpatient tertiary hospitals [39,41]. One study did not report LOS [43]. Overall, longer stays were observed in hospital-based populations compared with community-managed cases.

### 2.5. Comorbidities Among Older Adults Infected with RSV

Comorbidities were highly prevalent among the older adults included, with substantial variation in type and frequency across studies (Table 2). Across the included studies, cardiovascular conditions were commonly reported, including hypertension, chronic heart failure, and ischemic heart disease, among others [9,37,38,39,40,41,42,43]. Respiratory comorbidities were also frequently documented, particularly chronic obstructive pulmonary disease (COPD) and asthma [9,40,41]. Metabolic disorders, most notably diabetes mellitus, were reported in all studies that provided comorbidity data [9,43]. Several studies also reported neurologic conditions, including dementia, as well as immunocompromised states and chronic kidney disease [38,39,40,41,42]. A detailed overview of comorbidities across studies is presented in Table 2.

### 2.6. Antibiotic Prescription Rates Among RSV Patients

Among the RSV-infected older adults, the antibiotic prescription rate was consistently high across included studies, ranging from approximately 40% to 97.7% (Table 2). Antibiotic prescribing patterns are presented at the individual study level and are not pooled, given the substantial heterogeneity in study design, clinical setting, and diagnostic approach. Higher prescription rates were observed in hospital-based studies [9,39], while mixed inpatient–outpatient cohorts demonstrated variable rates [40,42]. Outpatient and emergency department cohorts reported comparatively lower but still substantial prescription rates [38,41,43]. One large administrative database study also reported high overall antibiotic exposure [37]. Study-specific prescription rates are detailed in Table 2.

### 2.7. Comparison of Antibiotic Prescription and Confirmed Bacterial Infection

**Figure 2:**
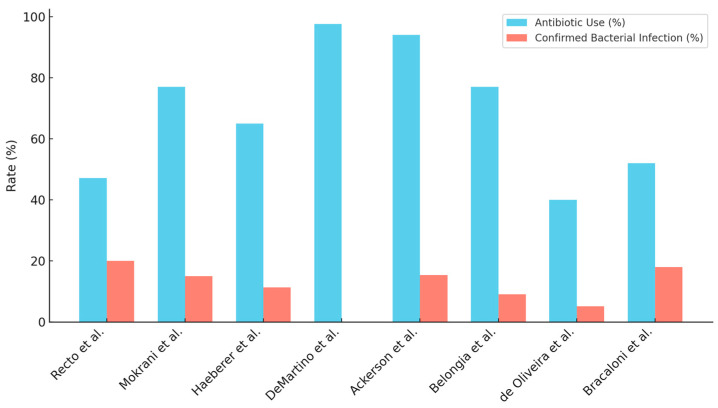
Comparison of antibiotic use (%) and confirmed bacterial infection (%) across older adult cohorts with confirmed RSV infection [9,38,39,40,41,42,43]. Bars represent study-specific rates. Absence of a red bar indicates that confirmed bacterial infection was not reported (DeMartino et al. [37]).

The relationship between antibiotic prescription and microbiologically confirmed bacterial infection is summarized in Table 2 and illustrated in Figure 2. Reported antibiotic prescription rates ranged from approximately 40% to 97.7%, whereas confirmed bacterial infections were reported at ≤20% across studies This pattern was consistent across inpatient, outpatient, and mixed clinical settings. One large administrative database study reported the highest antibiotic prescription rate but did not provide corresponding data on microbiologically confirmed bacterial infections [37]. Figure 2 presents the distribution of prescribing frequency in relation to confirmed infection rates across cohorts.

### 2.8. Clinical and Diagnostic Drivers of Antibiotic Prescription

Table 2 summarizes reported indications for prescribing antibiotics alongside antibiotic use, confirmed bacterial infection rates, hospitalization proportions, and LOS across the included studies. Several clinical and diagnostic factors were reported to be associated with antibiotic initiation in older adults infected with RSV. The underlying reasons were not always supported by microbiologically confirmed bacterial infection.

Empirical antibiotic prescription for suspected bacterial co-infection was frequently described, even in the absence of microbiological confirmation [9,38,39,40,42,43]. In one hospital-based cohort, 44% of antibiotic prescriptions were issued without collection of respiratory samples [39]. Radiological findings suggestive of pneumonia were frequently cited as indications for antibiotic therapy [9,38,39,40,41]. In one emergency department study, pneumonia-like findings were reported in 28.8% of patients, while microbiologically confirmed bacterial infection was documented in 20% [38]. Chest imaging was performed in more than 90% of patients in certain hospital-based studies [39,41], and in those cohorts, confirmed bacterial infection rates of less than 20% were reported in those cohorts were reported below 20%.

Elevated inflammatory markers, including C-reactive protein and leukocytosis, were described as factors associated with antibiotic initiation in several studies [38,40,41]. Respiratory symptoms and clinical signs were reported as triggers for antibiotic initiation across several studies [38,39,40,41,43]. Documented features included hypoxia, low oxygen saturation, dyspnea, wheezing, hypoxemia, and exacerbation of chronic respiratory conditions [38,39]. Additional symptom-based triggers included signs of lower respiratory tract infection, acute bronchitis, sinusitis, upper respiratory infection, productive cough, and increased sputum production [38,40,41,43]. Delays in RSV diagnostic testing preceding empirical antibiotic prescription were reported in one study [9]. The presence of chronic comorbidities, such as cardiovascular disease, chronic respiratory disease, diabetes, or renal impairment, was also reported to be associated with antibiotic prescribing decisions [9,39,41]. In one outpatient cohort, 15.1% of RSV-positive patients had initiated antibiotic therapy prior to clinical evaluation and RSV testing [43]. All reported drivers of antibiotic prescription are summarized in Table 2.

### 2.9. Antibiotic Initiation and Discontinuation in Relation to RSV Diagnosis

Across the included studies, antibiotics were frequently initiated empirically before diagnostic confirmation of RSV. In an ED cohort, early antibiotic initiation was reported in the context of delayed RT-PCR results and clinical suspicion of bacterial infection in febrile patients [38]. In a large multicenter hospital study, the mean time to a positive RSV result was 2.5 days, during which treatment decisions were based on clinical symptoms rather than confirmed virologic findings [9]. In a primary care setting, 15.1% of patients had already initiated antibiotics prior to clinical evaluation and nasopharyngeal sampling [43].

Data on antibiotic discontinuation following RSV confirmation were limited. A Spanish cohort study reported that antibiotics were often continued despite confirmation of viral infection [40]. In a French hospital-based study, 28% of patients with negative respiratory samples received antibiotics for more than seven days, with a mean excess duration of 3.6 days [39]. None of the included studies provided quantitative data on the proportion of patients whose antibiotics were discontinued after RSV confirmation.

### 2.10. Methodological Quality of Included Studies

Risk of bias was evaluated using the Newcastle–Ottawa Scale (NOS), and detailed scoring for each study is presented in Table 3. Overall, five studies were classified as high quality (NOS score 8–9/9), and three studies were rated as moderate quality (score 6–7/9).

Across moderate-quality studies, lower scores were primarily observed in the selection and comparability domains, reflecting a retrospective design, limited adjustment for confounding factors, or incomplete outcome reporting. Studies classified as high quality achieved full scores in most selection and outcome domains, frequently supported by clear case definitions and molecular confirmation of RSV infection and excellent follow-up rates. All NOS domain-specific scores are presented in Table 3.

## 3. Discussion

This review is the first to systematically assess antibiotic prescribing in older adults with RSV. Across studies, antibiotic use was consistently high (40.0–97.7%), while rates of confirmed bacterial infection rarely exceeded 20%. Most studies relied on molecular confirmation. The highest prescribing rate was reported in an administrative database using ICD codes, which may have resulted in an overestimation of RSV-related antibiotic exposure [37]. Population-level data indicate that RSV contributes to 2.1% of all antibiotic prescriptions and 4.3% of respiratory antibiotic prescriptions in primary care, particularly among adults ≥ 65 years [44].

These findings indicate that RSV likely contributes substantially to antibiotic exposure in older adults, largely driven by diagnostic uncertainty and perceived clinical risk [9]. Similar patterns have been observed in the case of other viral respiratory infections, including influenza and SARS-CoV-2, where high rates of empirical antibiotic use occur despite low rates of confirmed bacterial co-infection [9,39,40]. This highlights diagnostic uncertainty and concern for deterioration as key drivers across viral respiratory diseases.

Several included studies used multiplex PCR assays capable of detecting multiple respiratory viruses; however, viral co-infections were inconsistently reported and described only in a small proportion of patients (approximately 1–14%) [9,38,39,42,43]. Importantly, none of the studies analyzed their impact on antibiotic prescribing or outcomes, and some excluded co-infected patients [9,38]. As a result, the influence of viral co-infections on antibiotic use could not be assessed. Empirical therapy may still be justified in selected high-risk patients when bacterial infection cannot be excluded at presentation.

### 3.1. Diagnostic Uncertainty and Clinical Mimicry

RSV remains underdiagnosed in older adults due to inconsistent testing, and its non-specific symptoms often mimic bacterial pneumonia, prompting empirical antibiotic prescription [12,39,41,45]. Across studies, rates of antibiotic prescription remained high despite low rates of microbiologically confirmed bacterial infection. This finding aligns with external data showing that 61% of RSV-positive adults in one study received antibiotics, while only 8% had radiologically confirmed pneumonia [46]. Empirical therapy frequently begins before virologic confirmation; in one cohort, 14% of patients were already on antibiotics prior to admission [21]. Greater age, multimorbidity, and concern for severe outcomes, including pneumonia and ICU admission, further lower prescribing thresholds [9,38].

RSV testing alone does not reliably reduce antibiotic use, suggesting that diagnostic confirmation without structured stewardship may be insufficient to change current practices [47,48]. While empirical antibiotic therapy may be justified in selected high-risk older adults, particularly when bacterial infection cannot be safely excluded at initial presentation, the magnitude of the gap observed in this review suggests that precautionary prescribing alone does not explain current practices. Across the included studies, the antibiotic prescription rate ranged from 40% to 97.7%, whereas the rate of microbiologically confirmed bacterial infection did not exceed 20%, indicating a substantial discrepancy.

Although some level of empirical treatment is expected in vulnerable populations, this consistent imbalance suggests that a considerable proportion of antibiotic use may represent overuse rather than appropriate precaution. Limited evidence on reassessment and the lack of systematic reporting on antibiotic discontinuation following RSV confirmation indicate that initial empirical decisions are often not re-examined, potentially contributing to prolonged and unnecessary antibiotic exposure.

### 3.2. Over-Reliance on Non-Specific Indicators

Antibiotic initiation was frequently driven by non-specific clinical and laboratory indicators rather than microbiological confirmation. Radiologic findings such as pulmonary infiltrates commonly prompted treatment, although confirmed bacterial infection rates remained low; for example, suspected pneumonia led to an antibiotic prescription in 28.8% of patients in one cohort, while only 20% had a confirmed infection [38]. Elevated inflammatory markers (CRP, leukocytosis) and signs of respiratory distress, including hypoxia and dyspnea, were also associated with prescribing [38,39,40]. However, these features lack specificity and occur in both viral and bacterial infections.

In older adults with RSV, multimorbidity (e.g., chronic heart failure or COPD) may further confound clinical decision-making, as viral exacerbations of underlying conditions can mimic bacterial pneumonia [39]. This is compounded by non-specific laboratory and radiological findings, including elevated inflammatory markers and pneumonia-like infiltrates on imaging, which may be misinterpreted as bacterial infection [38,40]. In this context, diagnostic uncertainty with perceived patient vulnerability may contribute to increased empirical antibiotic prescribing despite low rates of confirmed bacterial co-infection.

Symptom-based cues such as productive cough and sputum production, along with perceived severity, further increased the prescribing rate, particularly in older patients less likely to undergo respiratory sampling [39,40,41,43]. Overall, reliance on non-specific indicators often outweighed microbiological evidence.

### 3.3. Impact of Diagnostic Delays

Delays in RSV diagnostic confirmation, ranging from 24 h to over 2.5 days, frequently resulted in empirical antibiotic initiation before viral etiology was established, with limited reassessment after confirmation. A Thai cohort reported that a 40.5 h median delay to PCR results was associated with prolonged and potentially unnecessary antibiotic use [49]. Similarly, 1–3-day delays in outpatient RT-PCR testing prompted antibiotic prescribing prior to RSV confirmation [50]. In a large hospital-based study, delayed diagnosis was linked to higher antibiotic exposure and a longer hospital stay; although 58.0% of patients received antibiotics, only 24.6% had a culture-confirmed bacterial infection [51]. Diagnostic delays exceeding 12 h were associated with longer treatment duration and prolonged LOS [51]. Together, these findings identify delayed RSV confirmation as a major driver of empirical antibiotic use.

### 3.4. Role of Underlying Comorbidities and Age

Multimorbidity was common among older adults infected with RSV, with high prevalences of hypertension (up to 73%), cardiovascular disease (up to 81%), COPD (up to 46%), and diabetes (up to 39%). These conditions increase vulnerability to complications and often lower the threshold for empirical antibiotic therapy [12,44,52]. Respiratory, cardiovascular, and metabolic comorbidities are associated with a higher hospitalization risk and more severe RSV outcomes [44,52]. Increasing age and comorbidity burden strongly influence perceived disease severity and treatment decisions [44]. Pulmonary disease, heart failure, and advanced age have been linked to greater hospitalization and antibiotic use [42,47], with each additional year of age associated with a 4% increased risk of a longer hospital stay [53].

However, despite these associations, no consistent relationship between cohort age distribution and antibiotic prescription rates was observed in this review, likely reflecting heterogeneity in settings. Instead, antibiotic prescribing appeared to be influenced more strongly by diagnostic uncertainty, illness severity, inflammatory markers, and local prescribing practices than age alone [12,39,41,44,51]. Symptom overlap between RSV and chronic disease exacerbations further complicates decision-making, underscoring the need for structured diagnostic and de-escalation strategies [42].

In older adults with respiratory infections such as RSV, multimorbidity and frailty may lower the threshold for initiating antibiotic therapy. RSV can exacerbate underlying chronic conditions, leading to complex clinical presentations that may mimic bacterial complications and contribute to diagnostic uncertainty. Clinicians may rely more heavily on clinical judgment and perceived illness severity, particularly in vulnerable patients, rather than awaiting definitive microbiological confirmation [39,43]. As a result, multimorbidity and frailty may contribute to precautionary antibiotic prescribing despite low rates of confirmed bacterial co-infection. These factors may also interact with non-specific clinical and diagnostic indicators, further complicating treatment decisions.

### 3.5. Influence of Clinical Setting on Antibiotic Prescription

Antibiotic prescribing varied by clinical setting and appeared to reflect perceived illness severity rather than confirmed bacterial infection. In inpatient cohorts, 77–94% of hospitalized RSV-positive adults received antibiotics [9,39], consistent with other hospital-based analyses showing that prescribing is driven primarily by clinical presentation and imaging rather than microbiological confirmation [52]. Substantial use was also observed in mixed inpatient–outpatient cohorts, with approximately 64% of hospitalized adults receiving antibiotics in one study despite limited radiologic pneumonia [47]. A U.S. Medicare database reported that antibiotics were prescribed in 97.7% of RSV-coded encounters, where diagnoses were based on administrative coding without bacterial confirmation [37]. Outpatient-only studies still reported considerable use (52–77%) [41,43,50]. Overall, empirical antibiotic prescription was common across care settings and more closely linked to perceived severity than to microbiological evidence.

These findings highlight differences between community and hospital settings. In community care, prescribing is predominantly influenced by diagnostic uncertainty and limited access to rapid testing, whereas in hospitals, it is driven by illness severity, radiological findings, and concern for complications [39,43]. Community-dwelling populations reflect less severe disease, while hospitalized patients are typically more complex and vulnerable. Accordingly, stewardship strategies differ, with greater emphasis on diagnostic support in primary care and on reassessment and de-escalation in hospital settings [54].

### 3.6. Hospitalization and LOS in Older Adults with RSV

Hospitalization rates ranged from 3% in primary care cohorts to nearly 100% in inpatient-only studies. Notably, hospitalization proportions in inpatient-only cohorts reflect study inclusion criteria and case selection rather than true admission probability and should not be interpreted as population-level hospitalization risk [39,40,42,43]. Higher admission rates were consistently associated with multimorbidity and respiratory compromise, particularly among patients with cardiovascular disease, COPD, diabetes, and chronic kidney disease [9,39,42].

The median LOS in inpatient cohorts ranged from 7 to 11 days compared with 3.5 days in community-managed cases [41]. Prolonged LOSs were associated with delayed diagnosis, greater severity, advanced age, and higher comorbidity burden [51,53]. Antibiotic exposure was not consistently linked to shorter hospitalization; some studies showed no association, while others reported longer stays among treated patients, likely reflecting confounding by illness severity rather than therapeutic benefit [27,53,55]. Overall, the LOS appeared to be primarily driven by patient complexity and disease severity rather than antibiotic use alone.

### 3.7. Management Failures and Antimicrobial Stewardship

While diagnostic uncertainty may justify initial empirical prescription, the persistence of therapy after RSV confirmation reflects an important lack of stewardship. Pre-hospital antibiotic exposure was common, with 28% of adults hospitalized for RSV having received antibiotics prior to admission [29]. Across studies, discontinuation following viral confirmation was inconsistent. In one cohort, 28% of adults with negative microbiology continued antibiotics for more than seven days [39], and other studies reported that 60–95% of RSV-hospitalized patients remained on systemic antibiotics despite minimal evidence of bacterial infection [27,52,56,57,58]. A Spanish cohort similarly observed frequent continuation after viral diagnosis, suggesting the absence of structured discontinuation practices [40].

Rapid molecular diagnostics alone did not reliably prevent unnecessary prescribing; in one study, over three-quarters of hospitalized adults received antibiotics, and nearly half were discharged while still on antibiotics, despite bacterial infection in fewer than 5% of cases [55]. These findings indicate that reassessment and de-escalation after viral confirmation remain insufficient, allowing continued antibiotic exposure in the absence of clear indication.

Importantly, the lack of systematic data on antibiotic discontinuation following RSV confirmation highlights a critical gap in antimicrobial stewardship practices. While initial empirical prescribing may be clinically justified, failure to reassess and discontinue antibiotics after viral diagnosis may contribute to unnecessary prolonged exposure. This underscores the need for structured stewardship strategies, including timely review of microbiological results, clear de-escalation protocols, and clinician support tools to guide antibiotic discontinuation after confirmed viral infection.

### 3.8. Implications for Clinical Practice and Public Health Strategy

Improving RSV management in older adults requires two immediate priorities: enhanced diagnostics and targeted prevention. Rapid molecular diagnostics that distinguish viral from bacterial infections are essential to reducing empirical antibiotic use, particularly in primary care. Preventive strategies are equally important. RSV treatment remains largely supportive, with antibiotics indicated only for confirmed bacterial infection [52,59,60]. Ribavirin is limited to high-risk or immunocompromised patients due to uncertain benefit [61]. The recent approval of RSV vaccines for adults ≥ 60 years, including RSVPreF3 (Arexvy, GlaxoSmithKline Biologicals, Rixensart, Belgium), RSVpreF (Abrysvo, Pfizer Inc., New York, NY, USA), and the mRNA-based vaccine mRESVIA (Moderna, Cambridge, MA, USA), provides a preventive opportunity to reduce severe disease, hospitalizations, and downstream antibiotic exposure. Antibiotic overuse drives antimicrobial resistance, which is projected to cause up to 10 million deaths annually by 2050 [62]. While increasing RSV vaccine uptake may contribute to reducing severe disease, hospitalizations, and potentially antibiotic exposure, these effects remain indirect and should be interpreted with caution [44].

### 3.9. Strengths and Limitations

Key strengths of this review include dual independent screening, formal risk-of-bias assessment, and the inclusion of predominantly molecularly confirmed RSV cases, with five out of eight studies rated high quality on the NOS. The exclusive focus on older adults enabled age-specific conclusions, and the inclusion of multiple care settings enhances generalizability. The comorbidity burden and other drivers of antibiotic prescription were also evaluated, offering insight into the mechanisms underlying potential unnecessary antibiotic use.

Limitations of this review include substantial heterogeneity in study design, clinical settings, patient populations, and diagnostic approaches; inconsistent reporting; and a lack of analysis of viral co-infections, which limited direct comparability across studies and constrained the strength of the conclusions. Differences in RSV confirmation methods, ranging from molecular diagnostics to administrative coding, as well as variations in healthcare settings and population characteristics, likely contributed to the variability in reported outcomes and prescribing patterns. Most studies were retrospective and hospital-based, potentially overestimating antibiotic use and hospitalization relative to community-dwelling populations.

Reporting of confirmed bacterial infections and antibiotic discontinuation was inconsistent, and not all studies systematically assessed microbiologically confirmed bacterial infection, limiting the evaluation of prescribing appropriateness. Generalizability is further limited by the predominance of studies from high-income countries and variable definitions of older adults (≥60 vs. ≥65 years). Additionally, reliance on observational designs and variable definitions of bacterial co-infection and antibiotic indications limit causal inference. The findings should therefore be interpreted cautiously and understood as indicative of overall trends rather than precise estimates.

## 4. Materials and Methods

This systematic review followed the Preferred Reporting Items for Systematic Reviews and Meta-Analyses (PRISMA) guidelines [63]. The completed PRISMA checklist is provided in the Supplementary Materials (Supplementary Table S1). The protocol of this systematic review was registered on the International Prospective Register of Systematic Reviews (PROSPERO) with the registration number CRD42024586905.

### 4.1. Inclusion and Exclusion

Studies were selected for inclusion based on the following predefined eligibility criteria:

- Population: Older adults aged ≥60 years. Studies including populations aged ≥65 years were also considered eligible. The age threshold used in each study is reported in Table 1.
- Exposure: RSV infection.
- Outcomes: Antibiotic prescription rates, confirmed bacterial infection, hospitalization, length of stay (LOS), and documented clinical drivers of antibiotic prescribing.
- Settings: Hospitals (inpatients, emergency departments), outpatient settings, and long-term care settings.
- Type of study: Observational study designs, including prospective and retrospective cohort studies, case–control studies, population-based studies, and registry or database analyses. Only articles published in peer-reviewed journals were included.
- Language: English.

Outcomes were classified as primary and secondary. Primary outcomes were the antibiotic prescription rate, confirmed bacterial infection rate, and hospitalization rate. Secondary outcomes included LOS (median days), documented indications for antibiotic prescription, and the prevalence of comorbidities among RSV-infected older adults. Standardized operational definitions are provided in Supplementary Methods S1.

### 4.2. Source and Search Strategy

We systematically searched PubMed/MEDLINE, Embase, Cochrane CENTRAL, Web of Science, and Scopus for studies published between January 2000 and August 2025. The initial search was conducted on 30 July 2024 and updated on 25 August 2025. The search strategy was developed in collaboration with an information specialist (SW) and tested and optimized using a set of twelve key articles. The search strategy combined controlled vocabulary (e.g., MeSH terms) and free-text keywords related to (1) respiratory syncytial virus (RSV), (2) antibiotic use, and (3) older or high-risk adults, including hospitalized patients. The search strategy excluded case reports, letters, and conference abstracts. Animal studies and studies focused exclusively on pediatric populations were also excluded. A date restriction was applied to include studies published from 2000 onwards. To identify further relevant studies, we screened the reference lists and citations of the included articles. Full search strategies for all databases are available in the Supplementary Methods S2.

### 4.3. Study Selection

All records were imported into EndNote 25, and duplicates were removed as described by Bramer et al. [64]. The deduplicated dataset was then imported into Rayyan for screening. Two reviewers (F.E and P.A) independently screened titles and abstracts against predefined eligibility criteria, followed by full-text screening. Disagreements were resolved by discussion or, when necessary, by a third reviewer (M.J.P). The study selection process is summarized in a PRISMA flow diagram (Figure 1), with the initial search diagram provided in the Supplementary Materials.

### 4.4. Data Extraction

Data extraction was conducted by one reviewer (F.E) using a standardized form and independently verified by a second reviewer (P.A). Extracted information included study characteristics, participant demographics, RSV diagnostic methods, antibiotic prescribing outcomes, confirmed bacterial infection rates, hospitalization outcomes, LOS, comorbidities, and reported clinical indications for antibiotic use. In studies examining multiple respiratory pathogens, only RSV-specific data were extracted. Viral co-infection data were not systematically extracted due to inconsistent reporting across studies and a lack of analysis in relation to the predefined outcomes. The full data extraction template is provided in Supplementary Table S2. All data were recorded exactly as reported in the original studies, without recalculation.

### 4.5. Risk of Bias Assessment

The methodological quality of the included observational studies was assessed independently by two reviewers (F.E. and P.A.) using the Newcastle–Ottawa Scale (NOS) [65]. The NOS is used for assessing the quality of non-randomized studies in meta-analyses. The NOS evaluates studies across three domains: selection of study groups (maximum 4 stars), comparability between groups (maximum 2 stars), and ascertainment of outcomes or exposures (maximum 3 stars). Discrepancies were resolved through discussion and consensus, with a third reviewer (M.J.P.) participating if needed. Based on total NOS scores, studies were classified as high quality (8–9), moderate quality (6–7), or low quality (≤5).

### 4.6. Data Synthesis

Due to substantial heterogeneity in study design, clinical settings, diagnostic methods, and outcome reporting, a quantitative meta-analysis or stratified analysis was not feasible. Instead, the findings were synthesized narratively at the individual study level using a descriptive comparison across studies. Antibiotic prescribing rates, confirmed bacterial infection rates, hospitalization outcomes, and clinical drivers of antibiotic use were summarized and compared across care settings and study populations. Tables and figures are used to present study-specific results and support structured comparison.

## 5. Conclusions

Empirical antibiotic use in older adults with RSV remains high despite low rates of microbiologically confirmed bacterial infection. Antibiotic prescribing is primarily driven by diagnostic uncertainty, illness severity, and multimorbidity rather than confirmed bacterial infection. Wider use of rapid molecular diagnostics and RSV vaccination may offer opportunities to reduce unnecessary antibiotic exposure, hospitalizations, and associated morbidity, although the impact of vaccination on antibiotic use remains indirect and requires further investigation. Future research should focus on prospective studies with standardized microbiological testing and systematic evaluation of antibiotic discontinuation to better assess prescribing appropriateness and long-term impact on antimicrobial resistance.

## Figures and Tables

**Table 1:** The demographic profiles of the included studies.

Reference	Year	*n* (RSV)	Study Design	Setting and Country	Mean Age	Age Inclusion Criterion (Years) ^a^	RSV Detection
Recto et al. [38]	2024	125	Single-center retrospective cohort study	Single-center; ED, Créteil, France	85.5	≥65 years	RT-PCR
Mokrani et al. [39]	2024	104	Retrospective monocentric Study	Single-center; Tertiary care hospital; inpatient wards & ICU, Paris, France	77.0	≥65 years	Multiplex RT-PCR
Haeberer et al. [40]	2024	706	Retrospective Cohort Study	Multicenter; 2 tertiary-care hospitals; inpatients & outpatients; Valladolid, Spain	79.5	≥60 years	PCR
DeMartino et al. [37]	2023	175,392	Retrospective, Longitudinal Cohort Study	Nationwide U.S. Medicare claims database; inpatient, outpatient & ED encounters; USA	79.0	≥60 years	ICD-9/10-CM codes
Ackerson et al. [9]	2019	645	Retrospective Observational Study	Multicenter; 15 community hospitals; inpatients; Southern California, USA	78.5	≥60 years	Multiplex PCR
Belongia et al. [41]	2018	243	Prospective observational cohort study	Community-based outpatient cohort; Wisconsin, USA;	72.2	≥60 years	Multiplex PCR
de Oliveira et al. [42]	2025	574	Retrospective cohort study	Single-center; public hospital center; inpatients & outpatients; Penafiel, Portugal	80.0	≥60 years	RT-PCR
Bracaloni et al. [43]	2024	33	Prospective pilot study	Multicenter; 17 general practitioners; primary care setting; outpatients; Italy	78.3	≥65 years	Multiplex RT-PCR

^a^ Age Inclusion Criterion (years) indicates the minimum age for inclusion in each study (≥60 or ≥65 years); ED = Emergency Department; ICD-9/10-CM = International Classification of Diseases, 9th/10th Revision, Clinical Modification; ICU = Intensive Care Unit; Multiplex PCR = polymerase chain reaction allowing simultaneous detection of multiple pathogens; RT-PCR = reverse transcription polymerase chain reaction.

**Table 2:** Summary of antibiotic use, bacterial infection rates, prescribing indications, hospitalization rates, and LOS in older adult patients infected with RSV across included studies.

Reference	Rate of Antibiotic Prescription in the Older Adults (%) ^a^	Confirmed Bacterial Infection Rate (%) ^b^	Indication for Antibiotic Prescription	Reported Comorbidities (Top Fives) ^c^	Rate of Hospitalization ^d^	LOS ^e^
Recto et al. [38]	47.2	20.0	Suspected bacterial co-infection, dyspnea, wheezing, oxygen saturation <90%, fever, high WBC/neutrophils, pneumonia on imaging, empiric antibiotics before PCR results, overprescription without radiologic evidence	Chronic heart failure (48.8%) COPD (19.2%) Diabetes (32.0%) Oncohematologic diseases (28.8%) Dementia (39.2%)	83.2	9.0
Mokrani et al. [39]	77.0	15.0	Respiratory infection management based on imaging, suspected bacterial infection, empirical treatment guided by subjective severity (including hypoxemia/respiratory distress), also without bacterial confirmation for existing comorbidities with older ages	Hypertension (63%) Heart disease (48%) COPD (39%) Diabetes (29%) Renal dysfunction (19%)	100.0	11.0
Haeberer et al. [40]	65.0	11.3	Empirically use for general diagnostic delays, suspected bacterial co-infection and signs of lower respiratory tract infection, pulmonary infiltrates on imaging, elevated inflammatory markers	Cardiovascular (81.1%) Endocrine/Metabolic (64.6%) COPD (46.1%) Immunocompromising conditions (42.9%) Neurologic conditions (30.1%)	90.0	9.0
DeMartino et al. [37]	97.7	Not explicitly mentioned	Not explicitly mentioned	Hypertension (73.0%) Hypoxia/dyspnea (52.8%) Chronic lung disease (44.1%) Pneumonia (41.3%) Congestive heart failure (35.5%)	25.7	8.0
Ackerson et al. [9]	94.1	15.4	Empirical antibiotic use for general diagnostic delays, suspected bacterial co-infection, pneumonia, or chronic disease exacerbation	Ischemic heart disease (32.2%) Congestive Heart Failure (35.3%) COPD (29.8%) Asthma (26.0%) Diabetes (38.9%)	100.0	7.0
Belongia et al. [41]	77.0	9.0	Pneumonia (imaging), acute bronchitis, acute sinusitis, URI, productive cough, increased sputum, abnormal chest findings, presence of comorbidities like COPD/CHF, routine hospital practice	COPD (15%) Asthma (14–20%) Congestive heart failure (7–9%) Diabetes (21%) Immunocompromised (6%)	12.0	3.5
de Oliveira et al. [42]	40.0	5.1	Suspected or confirmed co-bacterial infection	Hypertension (73%) Heart failure (38%) Diabetes (34%) COPD (24%) CKD (17%)	51.2	10.0
Bracaloni et al. [43]	52.0	18.0	Empirical use, increased frequency of prescribing associated with productive cough, presence of chronic comorbidities, or confirmed bacterial co-infection	Vascular (69.7%) COPD (24.2%) Diabetes (15.1%)	3.0	Not mentioned

^a^ Antibiotic prescription rate refers to the proportion of RSV-infected older adult patients who received at least one systemic antibiotic during the study period. ^b^ Confirmed bacterial infection rate refers to microbiologically proven bacterial co-infection (e.g., positive blood culture, sputum culture), as reported by each study. ^c^ Only the five most frequently reported comorbidities per study are shown. Percentages refer to the prevalence of each comorbidity within the RSV-positive older adult cohort of the respective study, as reported by the authors. ^d^ Rate of hospitalization indicates the percentage of RSV-infected patients who were admitted to the hospital for inpatient care. ^e^ Length of stay is reported in days and reflects the median value where specified. COPD = Chronic Obstructive Pulmonary Disease; CKD = Chronic Kidney Disease; CHF = Congestive Heart Failure.

**Table 3:** Newcastle–Ottawa Scale Quality Assessment of Included Studies.

References	Selection (Max 4)	Comparability (Max 2)	Outcome (Max 3)	Total (Max 9)	Quality Rating
Recto et al. [38]	4	2	3	9	High
Mokrani et al. [39]	3	2	3	8	High
Haeberer et al. [40]	4	2	3	9	High
De Martino et al. [37]	2	2	3	7	Moderate
Ackerson et al. [9]	4	2	3	9	High
Belongia et al. [41]	4	0	2	6	Moderate
de Oliveira et al. [42]	3	1	2	6	Moderate
Bracaloni et al. [43]	4	2	3	9	High

## Data Availability

The data presented in this study are included in the article and its Supplementary Materials. Further inquiries can be directed to the corresponding author.

## Supplementary Materials

The following supporting information can be downloaded at: https://www.mdpi.com/article/10.3390/antibiotics15050514/s1. Table S1: PRISMA 2020 checklist; Table S2: Data extraction form; Supplementary Methods S1: Outcome definitions and measurement; Supplementary Methods S2: Full search strategy.

## Abbreviations

The following abbreviations are used in this manuscript:

RSV	Respiratory syncytial virus
AMR	Antimicrobial resistance
LOS	Length of stay
NOS	Newcastle–Ottawa Scale
PRISMA	Preferred Reporting Items for Systematic Reviews and Meta-Analyses
