# Supplementary material for: Exploring the Link Between RSV Infection and Antibiotic Prescriptions in Older Adults: A Systematic Review
Source: Antibiotics (Basel). 2026 May 19;15(5):514. doi: 10.3390/antibiotics15050514 (PMC13203311; doi:10.3390/antibiotics15050514)
Supplement: Supplementary file 1 [file antibiotics-15-00514-s001.zip › antibiotics-4296210-supplementary.pdf]

Supplementary Methods S1. Outcome Definitions and Measurement

Supplementary Methods S2. Full Search Strategy

Supplementary Table S1. PRISMA 2020 Checklist

Supplementary Table S2. Data Extraction Form

Supplementary Methods S1. Outcome Definitions and Measurement

The primary and secondary outcomes of this systematic review were defined a priori to ensure consistency across heterogeneous observational studies.

Antibiotic prescription rate

Antibiotic prescription rate was defined as the proportion of RSV-positive older adult patients who received at least one systemic antibacterial agent during the study period, as reported by each included study. This included empirical or targeted antibiotic therapy administered in inpatient, outpatient, emergency department, or primary care settings. When studies reported multiple time points or antibiotic courses, the overall proportion of patients receiving any antibiotic treatment was extracted.

Confirmed bacterial infection

Confirmed bacterial infection referred to microbiologically documented bacterial infection, based on positive culture results (e.g., blood, sputum, or respiratory samples) or other laboratory confirmation, as defined by the original study authors. When studies reported both suspected and confirmed infections, only microbiologically confirmed cases were extracted.

Hospitalization rate

Hospitalization rate was defined as the proportion of RSV-infected older adults admitted to an inpatient facility for acute care. In studies restricted to hospitalized cohorts, the hospitalization rate was considered 100% by design. For mixed cohorts, the proportion of admitted patients among RSV-positive cases was recorded.

Length of stay (LOS)

Length of stay was defined as the duration of hospitalization in days among admitted RSV-positive patients. Median length of stay was extracted when reported. If only mean values were provided, these were recorded as reported by the study authors.

Comorbidities

Reported comorbidities were extracted as presented in the original studies. When available, the most frequently reported chronic conditions were documented, including cardiovascular disease, chronic respiratory disease, diabetes mellitus, neurologic disorders, and immunocompromised states. No attempt was made to standardize comorbidity definitions across studies.

Indications for antibiotic prescription

Documented clinical or diagnostic reasons for antibiotic prescribing were extracted verbatim from each study. These included suspected bacterial infection, radiologic findings suggestive of pneumonia, abnormal inflammatory markers, respiratory distress, symptom-based triggers, or presence of chronic comorbidities.

All outcome data were extracted as reported in the original studies without recalculation or reinterpretation.

Supplementary Methods S2. Full Search Strategy

We used the following list of key articles to draft, test and validate the search strategies (n=12).
PMIDs 37107150 34646942 30452608 19071185 38507143 35474419 30707378 35509398 12757561 30619907 31280089 36197575

MEDLINE (PubMed)
Concepts: 1.RSV-infection 2. Antibiotics 3. Elderly

("Respiratory Syncytial Virus Infections"[Mesh] OR "Respiratory Syncytial Virus, Human"[Mesh] OR "Respiratory Syncytial Virus Vaccines"[Mesh] OR
"respiratory syncytial virus*"[tiab] OR "rsv"[tiab] OR "rs virus"[tiab] OR
("respiratory"[ti] AND ("viral"[tiab] OR "virus*"[tiab] OR "non-bacterial"[tiab] OR "nonbacterial"[tiab] OR "prescri*"[ti] OR "inapprop*"[ti] OR "overus*"[ti] OR "misus*"[ti] OR "antibiotic*"[ti]) AND "infection*"[ti]) OR "nb arti"[tiab])
AND
("Anti-Bacterial Agents"[Mesh] OR "Drug Resistance, Microbial"[Mesh] OR "Anti-Bacterial Agents" [Pharmacological Action] OR "Antimicrobial Stewardship"[Mesh] OR "antibiotic*"[tiab] OR (("antimicrob*"[tiab] OR "anti-microb*"[tiab] OR "antibacter*"[tiab] OR "anti-bacter*"[tiab]) AND ("prescri*"[tiab] OR "inappropriat*"[tiab] OR "resistan*"[tiab] OR "overus*"[tiab] OR "misus*"[tiab]))
OR
"Respiratory Syncytial Virus Infections"[Majr] OR "Respiratory Syncytial Virus, Human"[Majr] OR "respiratory syncytial virus"[ti] OR "rsv"[ti])
AND
("Aged"[Mesh] OR "Middle Aged"[Mesh] OR "Age Factors"[Mesh] OR "Frailty"[Mesh] OR ("Hospitalization"[Mesh:NoExp] AND "Adult"[Mesh]) OR "elder*"[tiab] OR "frail*"[tiab] OR "ageing"[tiab] OR "older population*"[tiab] OR "older resident*"[tiab] OR "older adult*"[tiab] OR "older person*"[tiab] OR "older patient*"[tiab] OR "older adults"[tiab:~3] OR "older persons"[tiab:~3] OR "older patients"[tiab:~3] OR "older individuals"[tiab:~3] OR "older men"[tiab:~3] OR "older women"[tiab:~3] OR "old adult*"[tiab] OR "old person*"[tiab] OR "old patient*"[tiab] OR "old individual*"[tiab] OR "geriatric*"[tiab] OR "very old"[tiab] OR "oldest old"[tiab] OR "octogenarian*"[tiab] OR "nonagenarian*"[tiab] OR "centenarian*"[tiab] OR "65 older"[tiab:~2] OR "60 older"[tiab:~2] OR "over 65"[tiab:~2] OR "over 60"[tiab:~2] OR "older age"[tiab:~2] OR "older ages"[tiab:~2] OR "old age"[tiab:~2] OR "high risk adults"[tiab:~2] OR ("hospitali*"[tiab] AND "adult*"[tiab]) OR "hospitali* patient*"[tiab] OR (("Anti-Bacterial Agents"[Majr] OR "Drug Resistance, Microbial"[Majr] OR "Anti-Bacterial Agents" OR "Antimicrobial Stewardship"[Majr] OR "antibiotic*"[ti]) AND ("Respiratory Syncytial Virus Infections"[Majr] OR "Respiratory Syncytial Virus, Human"[Majr] OR "respiratory syncytial virus*"[ti] OR "rsv"[ti] OR "rs virus"[ti])))
AND
(2000:2026[pdat])
NOT
(("Animals"[Mesh] NOT "Humans"[Mesh]) OR (("Child"[Mesh] OR "Infant"[Mesh]) NOT "Adult"[Mesh]) OR ((child*[ti] OR pediatr*[ti] OR paediatr*[ti] OR neonat*[ti] OR infan*[ti]) NOT (adult*[ti] OR elder*[ti])) OR "Case Reports" [pt] OR "Letter" [pt])

Embase (embase.com)

('respiratory syncytial virus infection'/exp OR 'Human respiratory syncytial virus'/exp OR 'respiratory syncytial virus vaccine'/exp/mj OR 'viral respiratory tract infection'/mj OR
('respiratory syncytial virus*' OR 'rsv' OR 'rs virus' OR 'nb arti'):ab,ti,kw OR

(‘respiratory’:ti AND ‘infection*’:ti AND (‘viral’:ab,ti,kw OR ‘virus’:ab,ti,kw OR ‘nonbacterial’:ab,ti,kw OR ‘non bacterial’:ab,ti,kw OR ‘prescri*’:ti OR 'inapprop*':ti OR 'overus*':ti OR 'misus*':ti OR 'antibiotic*':ti)))

AND
('antibiotic agent'/exp OR 'antibiotic resistance'/exp OR 'antibiotic therapy'/exp OR 'antimicrobial stewardship'/exp/mj OR ‘antibiotic*’:ab,ti,kw OR ((‘antimicrob*’ OR ‘anti-microb*’ OR ‘antibacter*’ OR ‘anti-bacter*’) AND (‘prescri*’ OR ‘inappropriat*’ OR ‘resistan*’ OR ‘overus*’ OR ‘misus*’)):ab,ti,kw OR
'respiratory syncytial virus infection'/exp/mj OR 'Human respiratory syncytial virus'/exp/mj OR (‘respiratory syncytial virus’ OR ‘rsv’):ti)
AND
('aged'/exp OR 'middle aged'/exp OR 'age'/de OR 'senescence'/exp OR 'frailty'/exp OR 'aged hospital patient'/de OR (('hospitalization'/de OR 'hospital patient'/de) AND 'adult'/de) OR
(‘elder*’ OR ‘frail*’ OR ‘ageing’ OR ((‘old’ OR ‘older’) NEXT/3 (‘adult*’ OR ‘persons’ OR ‘patient*’ OR ‘men’ OR ‘women’ OR ‘resident*’ OR ‘population*’ OR ‘individual*’)) OR ‘geriatric*’ OR ‘very old’ OR ‘oldest old’ OR ‘octogenarian*’ OR ‘nonagenarian*’ OR ‘centenarian*’ OR ((‘65’ OR ‘60’) NEXT/2 ‘older’) OR (‘over’ NEXT/2 (‘60’ OR ‘65’)) OR ((‘old’ OR ‘older’) NEXT/2 (‘age’ OR ‘ages’)) OR (‘high risk’ NEAR/2 ‘adults’) OR (‘hospitali*’ NEAR/3 (‘adult*’ OR ‘patient*’))**)**:ab,ti,kw OR
(('antibiotic agent'/exp/mj OR 'antibiotic resistance'/exp/mj OR ‘antibiotic*’:ti) AND ('respiratory syncytial virus infection'/exp/mj OR 'Human respiratory syncytial virus'/exp/mj OR (‘respiratory syncytial virus*’ OR ‘rsv’ OR ‘rs virus’):ti)))
AND
[2000-2025]/py
NOT
(('animal'/exp NOT 'human'/exp) OR ('child'/exp NOT 'adult'/exp) OR ((child* OR pediatr* OR paediatr* OR neonat* OR infan*) NOT (adult* OR elder*)):ti OR 'case report'/exp OR 'letter'/exp OR 'conference abstract'/exp)

Web of Science Core Collection

(TS=(“respiratory syncytial virus*” OR “rsv” OR “rs virus” OR “nb arti”) OR
(TI=(“respiratory” AND “infection*”) AND TS=(“viral” OR “virus” OR “nonbacter*” OR “non-bacter*”))
OR
(TI=(“respiratory” AND “infection*”) AND TI=(inapprop*” OR “overus*” OR “misus*” OR “prescri*)))
AND
(TS=(“antibiotic*” OR ((“antimicrob*” OR “anti-microb*” OR “antibacter*” OR “anti-bacter*”) AND (“prescri*” OR “inappropriat*” OR “resistan*” OR “misus*” OR “overus*”))) OR
TI=(“respiratory syncytial virus” OR “rsv”))
AND
(TS=(“elder*” OR “frail*” OR “ageing” OR ((“old” OR “older”) NEAR/3 (“adult*” OR “persons” OR “patient*” OR “men” OR “women” OR “resident*” OR “population*” OR “individual*”)) OR “geriatric*” OR “very old” OR “oldest old” OR “octogenarian*” OR “nonagenarian*” OR “centenarian*” OR ((“65” OR “60”) NEAR/2 “older”) OR (“over” NEAR/2 (“60” OR “65”)) OR ((“old” OR “older”) NEAR/2 (“age” OR “ages”)) OR (“high risk” NEAR/2 “adults”) OR ((“adult*” OR “patient*”) NEAR/2 “hospitali*”)) OR TI=((“respiratory syncytial virus*” OR “rsv” OR “rs virus”) AND “antibiotic*”))
AND

(DT=(”Article” OR “Review”) AND PY=(2025 or 2024 or 2023 or 2022 or 2021 or 2020 or 2019 or 2018 or 2017 or 2016 or 2015 or 2014 or 2013 or 2012 or 2011 or 2010 or 2009 OR 2008 or 2007 or 2006 or 2005 or 2004 or 2003 or 2002 or 2001 or 2000))
NOT
TI=((“animal” OR “animals” OR “rat” OR “rats” OR “mouse” OR “mice” OR “murine” OR “rodent*”) OR ((child* OR pediatr* OR paediatr* OR neonat* OR infan*) NOT (adult* OR elder*)))

Cochrane Library
([mh "Respiratory Syncytial Virus Infections"] OR [mh "Respiratory Syncytial Virus, Human"] OR [mh "Respiratory Syncytial Virus Vaccines"] OR

("respiratory syncytial" NEXT virus*):ti,ab OR rsv:ti,ab OR "rs virus":ti,ab OR

(respiratory:ti AND (viral:ti,ab OR virus*:ti,ab OR non-bacterial:ti,ab OR nonbacterial:ti,ab OR prescri*:ti OR inapprop*:ti OR overus*:ti OR misus*:ti OR antibiotic*:ti) AND infection*:ti) OR "nb arti":ti,ab)

AND

([mh "Anti-Bacterial Agents"] OR [mh "Drug Resistance, Microbial"] OR "Anti-Bacterial Agents" OR [mh "Antimicrobial Stewardship"] OR antibiotic*:ti,ab OR ((antimicrob*:ti,ab OR anti-microb*:ti,ab OR antibacter*:ti,ab OR anti-bacter*:ti,ab) AND (prescri*:ti,ab OR inappropriat*:ti,ab OR resistan*:ti,ab OR overus*:ti,ab OR misus*:ti,ab)) OR

[mh "Respiratory Syncytial Virus Infections"] OR [mh "Respiratory Syncytial Virus, Human"] OR "respiratory syncytial virus":ti OR rsv:ti)

AND

([mh Aged] OR [mh "Middle Aged"] OR [mh "Age Factors"] OR [mh Frailty] OR ([mh ^Hospitalization] AND [mh Adult]) OR elder*:ti,ab OR frail*:ti,ab OR ageing:ti,ab OR ("older" NEXT population*):ti,ab OR ("older" NEXT resident*):ti,ab OR ("older" NEXT adult*):ti,ab OR ("older" NEXT person*):ti,ab OR ("older" NEXT patient*):ti,ab OR ("older" NEXT individual*):ti,ab OR "older men":ti,ab OR "older women":ti,ab OR ("old" NEXT adult*):ti,ab OR ("old" NEXT person*):ti,ab OR ("old" NEXT patient*):ti,ab OR ("old" NEXT individual*):ti,ab OR geriatric*:ti,ab OR "very old":ti,ab OR "oldest old":ti,ab OR octogenarian*:ti,ab OR nonagenarian*:ti,ab OR centenarian*:ti,ab OR "over 65":ti,ab OR "over 60":ti,ab OR "older age":ti,ab OR "older ages":ti,ab OR "old age":ti,ab OR "high risk adults":ti,ab OR (hospitali*:ti,ab AND adult*:ti,ab) OR (hospitali* NEXT patient*):ti,ab OR (antibiotic*:ti AND (rsv:ti OR "rs virus":ti)))
NOT
((child*:ti OR pediatr*:ti OR paediatr*:ti OR neonat*:ti OR infan*:ti) NOT (adult*:ti OR elder*:ti))
with Publication Year from 2000 to 2025, in Trials

Scopus

(TITLE-ABS-KEY(“respiratory syncytial virus*” OR “rsv” OR “rs virus” OR “nb arti”) OR (TITLE(“respiratory” AND “infection*”) AND TITLE-ABS-KEY(“viral” OR “virus” OR “nonbacter*” OR “non-bacter*”)) OR

(TITLE("respiratory" AND "infection*") AND TITLE(inapprop*" OR "overus*" OR "misus*" OR "prescri*)))
AND
(TITLE-ABS-KEY("antibiotic*" OR (("antimicrob*" OR "anti-microb*" OR "antibacter*" OR "anti-bacter*") AND ("prescri*" OR "inappropriat*" OR "resistan*" OR "misus*" OR "overus*"))) OR TITLE("respiratory syncytial virus" OR "rsv"))
AND
(TITLE-ABS-KEY("elder*" OR "frail*" OR "ageing" OR (("old" OR "older") W/3 ("adult*" OR "persons" OR "patient*" OR "men" OR "women" OR "resident*" OR "population*" OR "individual*")) OR "geriatric*" OR "very old" OR "oldest old" OR "octogenarian*" OR "nonagenarian*" OR "centenarian*" OR (("65" OR "60") W/2 "older") OR ("over" W/2 ("60" OR "65")) OR (("old" OR "older") W/2 ("age" OR "ages")) OR ("high risk" W/2 "adults") OR (("adult*" OR "patient*") W/2 "hospitali*")) OR TITLE(("respiratory syncytial virus*" OR "rsv" OR "rs virus") AND "antibiotic*"))
AND NOT TITLE("animal" OR "animals" OR "rat" OR "rats" OR "mouse" OR "mice" OR "murine" OR "rodent*")
AND NOT TITLE((child* OR pediatr* OR paediatr* OR neonat* OR infan*) AND NOT (adult* OR elder*))
Filters: 2000-2026; document type Article, Review

Supplementary Table S1. PRISMA 2020 Checklist

Supplementary Table S1 presents the completed PRISMA 2020 checklist for this systematic review. The checklist documents adherence to the Preferred Reporting Items for Systematic Reviews and Meta-Analyses (PRISMA) guidelines and indicates the manuscript sections in which each reporting item is addressed. This checklist is provided to ensure transparency and completeness of reporting.

Section	Item	PRISMA Item Description	Location in Manuscript
Title	1	Identify the report as a systematic review	Title page
Abstract	2	Structured abstract summarizing objectives, methods, results, and conclusions	Abstract
Introduction	3	Rationale for the review	Introduction
Introduction	4	Objectives or research questions	Introduction (last paragraph)
Methods	5	Eligibility criteria	Methods – Inclusion and Exclusion
Methods	6	Information sources	Methods – Source and Search Strategy
Methods	7	Search strategy	Methods + Supplementary Methods S2
Methods	8	Selection process	Methods – Study Selection
Methods	9	Data collection process	Methods – Data Extraction
Methods	10	Data items	Methods – Data Extraction and Outcome Definitions + Supplementary Methods S1 + Supplementary Table S2
Methods	11	Risk of bias assessment	Methods – Risk of Bias Assessment
Methods	12	Effect measures	Not applicable (descriptive synthesis; no quantitative effect measures)
Methods	13	Synthesis methods	Methods – Data Synthesis (narrative synthesis)
Methods	14	Reporting bias assessment	Not applicable (no meta-analysis performed)
Methods	15	Certainty assessment	Not applicable
Results	16	Study selection	Results – Study Selection + Figure 1
Results	17	Study characteristics	Results – Characteristics of Included Studies+ Table 1
Results	18	Risk of bias in studies	Results – Risk of Bias Assessment+ Table 3

Results	19	Results of individual studies	Results section + Tables 1-2
Results	20	Results of syntheses	Results section (narrative synthesis)
Results	21	Reporting biases	Not applicable
Results	22	Certainty of evidence	Not applicable
Discussion	23	Discussion of results	Discussion
Discussion	24	Limitations of evidence	Discussion– Strengths and Limitations
Discussion	25	Interpretation and implications	Discussion– Implications for Clinical Practice and Public Health
Other	26	Registration and protocol	Methods (PROSPERO registration)
Other	27	Support/funding	Funding Section
Other	28	Competing interests	Conflicts of Interest
Other	29	Availability of data/materials	Data Availability Statement

Supplementary Table S2. Data Extraction Form

Supplementary Table S2 shows the standardized data extraction form used to collect information from the included studies. The form was developed a priori and included predefined variables related to study characteristics, participant demographics, RSV diagnostic methods, antibiotic prescribing outcomes, bacterial infection rates, hospitalization outcomes, comorbidities, and clinical factors influencing antibiotic use.

Data were extracted exactly as reported in the original studies without recalculation. The standardized format ensured consistency across reviewers and facilitated structured comparison of heterogeneous observational studies.

Domain	Extracted Variable	Description / Measurement
Study identification	Authors	First author
Study identification	Year of publication	Year the study was published
Study identification	Journal	Journal name
Study characteristics	Study design	Observational study design (e.g., cohort, retrospective, prospective)
Study characteristics	Setting	Clinical setting (inpatient, outpatient, ED, primary care)
Study characteristics	Country	Country where the study was conducted
Population characteristics	Sample size (RSV cases)	Number of RSV-positive older adults included
Population characteristics	Age	Mean or median age and age inclusion criteria
Population characteristics	Comorbidities	Reported chronic conditions in study population
Diagnostic methods	RSV detection method	PCR, RT-PCR, multiplex PCR, or administrative coding
Antibiotic outcomes	Antibiotic prescription rate	Proportion of RSV patients receiving antibiotics
Antibiotic outcomes	Indications for antibiotic use	Reported clinical reasons for prescribing
Infection outcomes	Confirmed bacterial infection rate	Microbiologically confirmed infection rates
Clinical outcomes	Hospitalization rate	Proportion of RSV patients admitted to hospital
Clinical outcomes	Length of stay (LOS)	Duration of hospitalization in days

Additional notes	Comments	Relevant methodological notes or clarifications
